# CMV promoter mutants with a reduced propensity to productivity loss in CHO cells

**DOI:** 10.1038/srep16952

**Published:** 2015-11-19

**Authors:** Benjamin Moritz, Peter B. Becker, Ulrich Göpfert

**Affiliations:** 1 Roche Pharmaceutical Research and Early Development, Large Molecule Research, Roche Innovation Center Penzberg, Germany; 2 Biomedical Center and Center for Integrated Protein Science Munich, Ludwig Maximilian University, Munich, Germany

## Abstract

The major immediate-early promoter and enhancer of the human cytomegalovirus (hCMV-MIE) is one of the most potent DNA elements driving recombinant gene expression in mammalian cells. Therefore, it is widely employed not only in research but also in large-scale industrial applications, e.g. for the production of therapeutic antibodies in Chinese hamster ovary cells (CHO). As we have reported previously, multi-site methylation of hCMV-MIE is linked to productivity loss in permanently transfected CHO cells lines. In particular, the cytosine located 179 bp upstream of the transcription start site (C-179) is frequently methylated. Therefore, our objective was to study whether mutation of C-179 and other cytosines within hCMV-MIE might lessen the instability of transgene expression. We discovered that the single mutation of C-179 to G can significantly stabilise the production of recombinant protein under control of hCMV-MIE in permanently transfected CHO cells.

For the expression of therapeutic proteins, such as immunoglobulins and other multimeric or glycosylated proteins, Chinese hamster ovary cells (CHO) are the preferred producers^1^. For economic reasons and in order to facilitate process upscaling, cell lines with high and stable productivity are desirable. Any significant decrease of productivity during scale-up constitutes a serious risk during cell line development^2^,^3^. In order to obtain high expression levels, the hCMV-MIE promoter and enhancer is widely used to drive recombinant gene expression in research and manufacturing^4^,^5^,^6^,^7^. Although hCMV-MIE provides high gene expression levels, decreased productivity has been reported when cultivation times are extended^8^,^9^,^10^. hCMV-MIE is prone to transcriptional silencing which is associated with DNA methylation^11^,^12^,^13^,^14^,^15^. Mammalian DNA is predominantly methylated at cytosine bases that are part of CpG dinucleotides^16^,^17^. Loss of activating histone modifications, or accumulation of repressive histone modifications, are often reported as early silencing events upstream of DNA methylation^18^,^19^. There are thirty-three CpG sites within 600 bp upstream of the hCMV-MIE transcription start site (Fig. 1a). In recombinant CHO cell lines with decreasing productivity, the cytosine at position −179 was found to be the most frequently methylated site. Additional methylation events are clustered at the 5′-end and the 3′-end of the promoter sequence, i.e. within the enhancer and core promoter region^12^,^13^. In particular, the cytosines at positions −508, −41 and −13 seem to be frequently methylated^12^. In order to check whether removal of CpG sites can stabilise hCMV-MIE driven gene expression, we point-mutated C-508, C-179 and C-41 in various combinations. The production of recombinant protein was significantly increased after prolonged cultivation of stably transfected CHO cells, if hCMV-MIE was engineered to bear the single C-179 to G mutation. Furthermore, the propensity of transfected cells to lose specific productivity over cultivation time was reduced.

## Results

**Figure 1:**
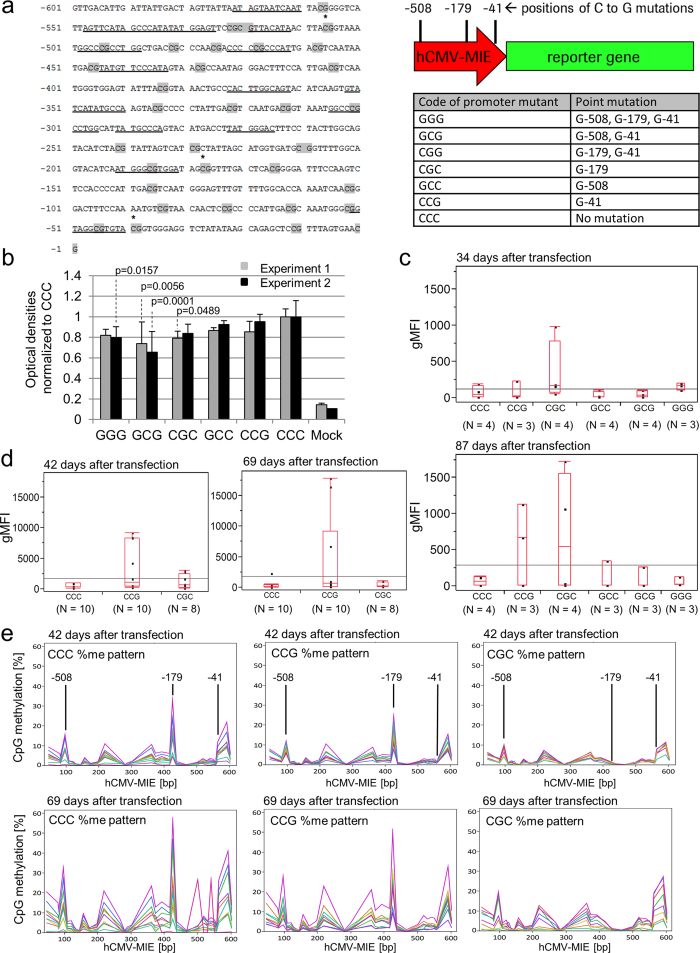
(**a**) hCMV-MIE sequence (left) and list of mutants (right). Positions where C to G mutations were introduced are marked with an asterisk. Transcription factor binding sites are underlined. CpG sites are highlighted in grey. hCMV-MIE variants are coded with a three letter identifier referring to the nucleotides at positions −508, −179 and −41. DNA positions are numbered relative to the transcription start site. **(b)** Transient expression of SEAP driven by hCMV-MIE mutants. Expression levels were normalised to unmutated hCMV-MIE (CCC). Error bars represent SD of eight biological replicates. Two independent experiments were performed. Potential effects of the hCMV-MIE mutations within the same experiment were tested with Tukey HSD (α = 0.05). Significant differences between the promoter mutants and CCC within the same experiment are marked with the respective p-value. **(c)** eGFP expression of permanently transfected CHO cell pools 34 days (upper panel) and 87 days (lower panel) after transfection. eGFP expression was quantified by FACS and the geometrical mean of the fluorescence intensity (gMFI) was plotted for each cell pool. The identity of the promoter variants and the number (N) of independent cell pools is indicated below the x-axis. The upper and the lower ends of the boxes represent the first and the third quartile of each group. The ends of the whiskers represent the lowest and highest values still within the 1.5fold interquartile range. The grey line indicates the overall mean of the gMFI values. **(d)** eGFP expression of permanently transfected CHO cell pools 42 days (left panel) and 69 days (right panel) after transfection; all further labelling is identical to (**c**). **(e)** Percentage methylation levels of hCMV-MIE variants at each CpG cytosine 42 days (upper panels) and 69 days (lower panels) after transfection. Methylation levels are plotted against DNA positions numbered 5′ to 3′. The positions of C-508, C-179, and C-41 are highlighted.

In order to monitor the effects of methylation-prone CpG sites from the enhancer, the proximal and the core promoter region, we chose to investigate not only C-179 but also the cytosines at positions −508 (C-508) and −41 (C-41). None of the selected CpG sites overlaps with known transcription factor binding sites in *Cricetidae* species. In order to maintain the overall GC content, we performed C to G transversions (Fig. 1a). Besides C-41, C-13 is the second cytosine in the core promoter region that seems to be frequently methylated. Because mutation of C-13 to G would create a new CpG site, we preferred to investigate C-41G. The mutated promoter variants were abbreviated with a three letter code, e.g. variant GCC has guanine instead of cytosine at position −508, whereas the cytosine bases at −179 and −41 are preserved. CCC is the unmutated control hCMV-MIE (Fig. 1a). Mutated or native promoters were introduced into various plasmids and placed upstream of different reporter genes.

In order to see whether the mutations affect the strength of the promoter, we performed two independent experiments by transiently transfecting reporter plasmids expressing secreted embryonic alkaline phosphatase (SEAP) into CHO-K1 cells and examined the expression of SEAP five days after transfection. The unmutated promoter was used as reference (Fig. 1b). Expression of SEAP from GGG, GCG, and CGC promoters was consistently about 20% lower than from the unmutated promoter, with minor variation between the replicate experiments. In general, the C to G mutations displayed a tendency toward modestly reduced promoter strength which, however, appeared to be acceptable.

Next, we asked whether hCMV-MIE promoter variants influence the expression stability of permanently transfected CHO cells during long term cultivation. For this purpose, we chose an eGFP reporter which enables the cytometric analysis of reporter gene expression at the single cell level. eGFP reporter plasmids were transfected into CHO-K1 cells, and three to four independent pools of permanently transfected cells were selected with methotrexate (MTX). These were cultivated over three months in the presence of selection agent MTX. Thirty-four and eighty-seven days after transfection, eGFP expression was measured by cytometry (two pools were lost between day 34 and 87 due to contamination). The geometrical means of the fluorescence intensity of 10,000 events per measurement were calculated for each cell pool and plotted for both time points (Fig. 1c). Thirty-four days after transfection there was little difference between the constructs. Only one CGC pool showed elevated expression levels. Eighty-seven days after transfection, two out of four pools transfected with CGC and two out of three pools transfected with CCG displayed distinctively higher eGFP values than all other pools, transfected either with the unmutated control or the remaining promoter variants. These observations suggested a stabilising effect of the single C-41G or C-179G mutations on eGFP expression. In order to consolidate this result, we repeated the experiment with both promoter variants and with a larger number of pools. We generated ten pools with CCG or CCC, respectively, and eight pools with CGC (two pools were lost due to contamination). The cells were cultivated as before and analysed by flow cytometry from day 42 to day 69 after transfection (Fig. 1d and Supplementary Fig. S1 online). After 42 days, eGFP expression was strongly increased in four CCG pools compared with the CCC control pools, whereas two CGC pools demonstrated a moderate increase in eGFP expression (Fig. 1d, left panel). After 69 days, three CCG pools still displayed high levels of eGFP, whereas eGFP expression of the CGC pools had dropped to CCC control levels (Fig. 1d, right panel). The differences between the groups were tested at each time point with the non-parametric Steel-Dwass test. The alpha level was set to 0.05. A significant difference was observed between CCG and CCC on day 56 after transfection (p = 0.0305; see Supplementary Fig. S1 online). Taken together, the results of Fig. 1c,d suggested that both mutations C-41G and C-179G can delay the loss of hCMV-MIE driven recombinant gene expression.

Mutations C-41G and C-179G completely inhibit DNA methylation at the respective site since guanine cannot be methylated. We wondered whether these mutations also affected methylation levels at other CpG sites within hCMV-MIE. Therefore, we measured the methylation levels of all CpG sites in the individual hCMV-MIE variants by bisulphite conversion coupled with next generation sequencing. To this end, we extracted genomic DNA from the cell pools of the second experiment 42 and 69 days after transfection and treated it with bisulphite to discriminate between methylated and non-methylated cytosine. hCMV-MIE DNA was amplified and sequenced with the Illumina MiSeq system (Fig. 1e). C-179 was most strongly methylated within all pools transfected with either CCC or CCG, confirming previous observations with the unmutated hCMV-MIE^12^. Furthermore, we observed a distinct methylation pattern that was very similar with all pools but differed in overall intensity. In particular, unmutated C-508 and C-41 were consistently among the most frequently methylated sites. The mutation of C-41 in promoter variant CCG, particularly the mutation of C-179 in variant CGC, appear to uniformly decrease the methylation of all CpG sites rather than change the methylation pattern. When we calculated the average methylation levels across all CpG sites for each pool, we detected lower overall methylation with the mutated promoter variants (see Supplementary Fig. S2 online). The difference was most pronounced with variant CGC but did not reach statistical significance with the Steel-Dwass test (α = 0.05). On average, methylation increased over time with all constructs.

Besides epigenetic promoter silencing, loss of transgene copies is a major cause of production instability in recombinant cells^20^. In order to estimate the relative contribution, we measured plasmid copy numbers by qPCR 42 and 69 days after transfection. Average plasmid copy numbers decreased over time, with no obvious difference between the constructs (see Supplementary Fig. S2 online). Interestingly, the distribution of copy numbers was shifted towards lower values in the CGC group. However, when we tested statistical significance with the Steel-Dwass test, the differences between the constructs did not prove to be significant (α = 0.05).

Pools of permanently transfected cells are highly heterogeneous mixtures of clones differing in transgene expression and growth rate. Highly productive clones tend to have a lower growth rate^21^. Therefore, low producing cells, many of which are present in the culture after transfection, almost inevitably overgrow the high producers sooner or later. In order to minimise this bias, we investigated the mutations C-179G and C-41G in clonal cell lines. Since we aimed to assess the effects on the production of a potential commercial product, we switched to an IgG-IL2 fusion protein as reporter. Thus, promoter variants containing both mutations either alone (CGC or CCG) or in combination (CGG) were inserted upstream of the light chain and the heavy chain gene of an IgG-IL2 expression plasmid.

Expression plasmids carrying either CCG, CGC, CGG or the unmutated hCMV-MIE were transfected into CHO-K1 cells, and stably transfected clones were isolated in 384-well plates. Thirty clonal cell lines per construct were randomly selected and expanded into six-well plates. After the cell lines had shown stable growth, we studied their productivity for approximately two months, from day 68 to day 134 after transfection. Some cell lines were lost due to contamination or reduced viability, yet 23 to 29 clones per construct were examined over the entire period of time.

**Figure 2:**
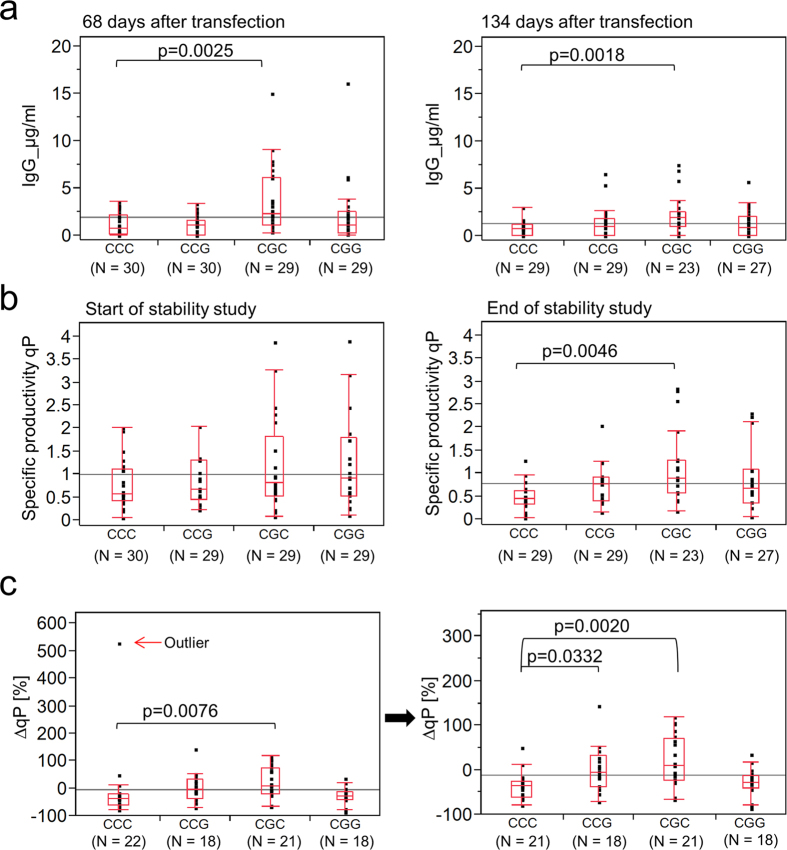
hCMV-MIE variants CGC and CCG stabilise the production of recombinant protein in clonal CHO cell lines. **(a)** IgG-IL2 titres of CHO clones 68 days (left panel) and 134 days (right panel) after transfection. **(b)** Specific productivity *qP* of CHO clones at the beginning (left panel) and end (right panel) of long-term cultivation. Early *qP* values were calculated by averaging the *qP* values of two early passages representing the two earliest data points (days 68–71 and days 83–86 after transfection). Late *qP* values were calculated by averaging the last three passages (days 124–127, days127–131, and days 131–134 after transfection) **(c)** Percentage change of specific productivities *∆qP* during long-term cultivation based on data shown in (**b**) with (left panel) and without (right panel) one outlier identified by jackknife analysis. Only clones producing detectable levels of product on day 68 were considered. (**a–c**) The identity of the promoter variants and the number (N) of independent cell pools is indicated below the x-axis. The upper and the lower ends of the boxes represent the first and the third quartile of each group. The ends of the whiskers represent the lowest and highest values still within the 1.5fold interquartile range. The grey line indicates the overall mean of the y-variable in the diagram. Significant differences (α = 0.05) between the groups according to the Steel-Dwass test are indicated by the p-values.

During the entire study we determined IgG-IL2 concentrations in cell culture supernatants and productivity per cell and day (specific productivity *qP*). At the beginning (day 68) and end (day 134) of the study, cell lines transfected with CGC showed significantly higher titres than the control transfectants (α = 0.05; Fig. 2a). Cell lines with remarkably high titres were also observed among the CGG transfectants on day 68, and among the CCG and CGG transfectants on day 134. When we compared specific productivities between cell lines transfected with different constructs, we made similar observations (Fig. 2b). In order to compensate for the imprecision in cell counting, we averaged the specific productivities *qP* of two passages in the beginning (day 68–71 and 83–86 after transfection representing the earliest available data points) and three passages at the end of long-term cultivation (day 124–127, day 127–131 and day131–134 after transfection). Significant differences between cell lines transfected with CGC or CCC were observed at the end of long-term cultivation (α = 0.05).

Higher titres and specific productivities of cell lines carrying G-179 or G-41 suggested a stabilising effect of these mutations on hCMV-MIE driven gene expression. In order to document this effect, we calculated the percentage alteration of *qP* during long-term cultivation for each individual cell line as the most appropriate measure of its production stability (Fig. 2c). Of course, this was feasible only with cell lines still producing IgG-IL2 on day 68 after transfection. One control transfectant increased its specific productivity by 500% and was identified as an outlier by jackknife outlier analysis (Fig. 2c, left plot). Therefore this cell line was excluded from further analysis (Fig. 2c, right plot). Cell lines transfected with either CGC or CCG were significantly more stable than control cell lines transfected with CCC (α = 0.05). Surprisingly, the double mutant CGG did not differ from the control.

## Discussion

In our study, we point-mutated the hCMV-MIE promoter and enhancer at three methylation-prone CpG sites in various combinations and studied the effect on long-term productivity of permanently transfected CHO cells. We investigated eGFP-expressing CHO pools and clonal cell lines secreting an IgG-IL2 fusion protein. In summary, we observed that the single point mutations C-41G and C-179G both had a positive impact on the maintenance of recombinant protein production in permanently transfected CHO cells. It is tempting to speculate that the effect of C-41G and C-179G is based on reduced silencing of hCMV-MIE since C-41 and C179 are among the most frequently methylated sites in hCMV-MIE. Furthermore, C-41G and C-179G did not increase the numbers of plasmid copies in transfected CHO pools or decrease the propensity of transfected CHO pools to lose plasmid copies. Importantly, these mutations did not adversely affect absolute expression levels as one might have expected from previous transient transfection experiments. Instead, the highest producers of each experiment were among those cells carrying a mutant promoter. The effect in CHO pools was not as distinctive as in clonal cell lines. This may be due to the higher variability and consequently the increased selection dynamics in CHO pools. In contrast to clonal cell lines, CHO pools contain various cells with different characteristics right after transfection^22^. Low producers which, for instance, integrated the foreign DNA at a less active genome site will gradually overgrow the high expressors and thereby mask the effect of differential promoter silencing, irrespective of the promoter variants. Consequently, the effects of hCMV-MIE mutations on gene expression can be assessed more reliably in clonal cell lines. As the C-179G mutation was more effective than the C-41G mutation in clonal cell lines, we conclude that G-179 is more potent than G-41 in maintaining recombinant protein production. This is not unexpected, since C-179 is by far the most frequently methylated site in wild-type hCMV-MIE, suggesting that this site plays a dominant role in silencing the hCMV-MIE.

We did not determine the plasmid copy numbers in the clonal lines and can, therefore, not formally exclude the possibility that the stabilising effect in clonal lines is due to increased transgene copies. We nevertheless consider such a scenario unlikely since the major difference at the DNA level between the studies with CHO pools and clonal cell lines is the reporter gene, which is unlikely to systematically affect copy numbers in the numerous clonal lines bearing C-41G or C-179G mutations. Certainly, studying isogenic clones with one single-copy integrand would facilitate the analysis since a loss of transgene copies or specific effects of the integration site would not overlay a potential epigenetic effect of the promoter variant^23^. Nevertheless, the random integration approach, which usually results in multicopy integrands at random sites, is still very common in the biopharmaceutical industry. Therefore, it is very relevant to study the effect of hCMV-MIE mutants in multiple copy cell lines and to demonstrate that they are beneficial in this context.

Interestingly, combined mutations neither enhanced expression stability in eGFP cell pools (e.g., GGG) nor in clonal cell lines expressing IgG-IL2 (CGG). This argues against the GC skew^24^ playing a role in stabilising hCMV-MIE driven gene expression, but in favour of a more complex mechanism. As we observed a very distinct methylation pattern at the hCMV-MIE promoter, it is tempting to speculate that a specific modulation of this pattern has a positive impact on hCMV-MIE expression stability, rather than the overall methylation levels. In this respect, it is important to note that unmethylated CpG-rich sequences can attract protein complexes which promote activating as well as inactivating methylation of histone H3^25^,^26^. As a consequence, the mutation of a CpG site in a specific context may even promote silencing. Apart from preventing specific methylation at the mutated site, C-41G and C-179G considerably reduced the methylation frequency of all CpG sites of hCMV-MIE in eGFP expressing CHO pools. This effect may contribute to a potential epigenetic stabilisation of hCMV-MIE driven gene expression.

Unmethylated CpG islands inserted upstream or within hCMV-MIE were shown to stabilise hCMV-MIE driven gene expression in CHO-K1^22^,^27^. However, the function of recombinant epigenetic regulatory elements can be affected by the vector context^28^. In this regard, more direct inhibition of silencing by mutation of methylation prone CpG sites is an attractive option. We demonstrated that the removal of a single CpG within the hCMV-MIE promoter can have a strong effect on expression levels and long-term stability. Previously, mutation of CpGs was shown to stabilise gene expression from the MSCV long terminal repeat in embryonic stem cells^29^. To our knowledge, point mutations of single methylation-prone promoter CpG sites have not been reported before. In agreement with recent findings for the IL6 gene promoter, our data suggest that individual CpG sites are of particular importance for the silencing of hCMV-MIE^30^. Clearly, more studies on copy numbers, DNA methylation as well as hCMV-MIE chromatin may help elucidate the underlying mechanism.

Stability of recombinant expression is a major prerequisite of reporter cell lines as well as commercial production cell lines. These cell lines are very often transfected with hCMV-MIE carrying recombinant DNA. A single point mutation is easily introduced in any DNA vector. Therefore, we believe that the hCMV-MIE variant CGC will be of great benefit for a broad community of users.

## Material and Methods

### Plasmids

Recombinant cells expressing either secreted embryonic alkaline phosphatase (SEAP), enhanced green fluorescence protein (eGFP), or a human IgG-IL2 fusion protein were generated by transient or stable transfection of CHO-K1 suspension cells with a plasmid expressing the reporter gene from a human major immediate-early promoter/enhancer fragment (hCMV-MIE). Light and heavy chain genes of IgG-IL2 were both located on the same plasmid and under control of the identical human hCMV-MIE variant. IL2 was fused to the immunoglobulin heavy chain. Point mutations were introduced into hCMV-MIE with the QuikChange Multi-Site-Directed Mutagnesis Kit (Agilent Technologies, Waldbronn, Germany) following the manufacturer’s protocol. The vectors also carry a sequence encoding murine dihydrofolate reductase (DHFR) under the control of the SV40 early promoter.

### Cultivation and transfection of cells

For transient expression of SEAP in CHO-K1, cells were transfected with circular DNA using the Nucleofector™ 96-well Shuttle™System and the Nucleofector Kit V (Lonza, Cologne, Germany). After transfection, the cells were seeded in 96-well plates and cultivated for five days. SEAP concentration in the culture medium was measured as described by Cullen and Malim^31^.

For permanent expression of eGFP or IgG-IL2, cells were transfected with linear DNA using the Nucleofector™2b Device and the Nucleofector Kit V (Lonza, Cologne, Germany). Stably transfected cells were either selected and further cultivated as pools in disposable 125 ml vented shake flasks or isolated as clonal cell lines in 384-well plates and expanded into 6-well plates. Cells were cultivated in a humidified atmosphere at 37 °C and 5% to 7% CO_2_. Shake flasks and 6-well plates were constantly agitated at 150 rpm/min or 120 rpm/min, respectively. Selection was performed in thymidine-free medium with 250 to 1600 nM methotrexate (MTX). Subsequently, the concentration of MTX was reduced to 250 nM MTX in all cultures. Every 3–4 days the cell cultures were diluted with fresh protein-free medium to a concentration of 2–3 × 10^5^ cells/ml. Density and viability of the cultures were measured either with a Cedex HiRes cell counter (F. Hoffmann-La Roche Ltd, Basel, Switzerland) or with a Cellavista cell imager (SynenTec, Münster, Germany). eGFP expression was examined by flow cytometry, whereas antibody titres were measured by ELISA.

### Analysis of eGFP and antibody expression

eGFP expression in CHO-K1 was examined before passaging the cells with a BD FACS Canto II flow cytometer (BD, Heidelberg, Germany). 10,000 events per sample were measured. Living cells were gated in a plot of forward scatter (FSC) against side scatter (SSC). The live cell gate was defined with untransfected cells and applied to all samples by employing the FlowJo 7.6.5 EN software (TreeStar, Olten, Switzerland). Fluorescence of eGFP was quantified in the FITC channel (excitation at 488 nm, detection at 530 nm), and the signals were transferred into a histogram. The geometrical mean of fluorescence intensity was calculated for each sample, and the geometrical means of promoter variants were compared to the unmutated promoter control.

The concentration of IgG-IL2 in cell culture supernatants was determined with an anti-human IgG sandwich ELISA. In brief, IgG-IL2 was captured from the cell culture fluid with an anti-human Fc antibody bound to a microtitre plate (Maxisorp, Roche) and detected with an anti-human Fc POD conjugate which binds to an epitope different from the capture antibody. The secondary antibody was quantified by chemoluminescence employing the BM Chemiluminescence ELISA Substrate (POD) (Roche, Penzberg, Germany). The concentration of IgG-IL2 in samples was extrapolated from a standard curve generated with purified IgG-IL2. From these data, the specific productivity *qP* (pg/cell/day) for an individual passage was calculated using equation 1:


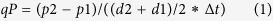


with *p2* and *p1* being the final and initial antibody concentrations, *d*2 and *d1* being the final and initial viable cell densities, and *∆t* the duration of the passage.

As a measure of production stability over a certain cultivation period, the percentage change of *qP* was calculated using equation 2:


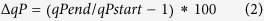


with *∆qP* being the percentage change in *qP*, *qPstart* being the specific productivity at the beginning and *qPend* being the specific productivity at the end of the cultivation period.

### Quantification of transgene copies

The number of transgene copies was determined by qPCR as described by Osterlehner *et al.*^12^, using forward primer 5′-TACATCAATGGGCGTGGATA-3′ and reverse primer 5′-AAGTCCCGTTGATTTTGGTG-3′, both binding inside hCMV-MIE.

### Methylation of hCMV-MIE

Genomic DNA was extracted from recombinant CHO pools and treated with bisulphite as described previously^12^. Bisulphite-converted hCMV-MIE DNA was amplified by PCR using primers 5′-GATATTGATTATTGATTAGTTATTAATAGTAATTAA-3′ and 5′-CAAATAAAAAAATCCCATAAAATCATATACTAA-3′ for amplicon 1 and primers 5′-TTAGTATATGATTTTATGGGATTTTTTTATTTG-3′ and 5′-TTCTAATACTAAACTCCTCTCCCAA-3′ for amplicon 2. Both amplicons were sequenced with the MiSeq system (Illumina, Inc., San Diego, USA). The concentration of amplified DNA was measured with the Qubit system (Life Technologies GmbH, Darmstadt, Germany). The library was generated according to the TruSeq Nano DNA Library Prep Guide (Part # 15041110 Rev. C, Illumina, Inc., San Diego, USA) using 100 ng of each amplified product. Library quality was checked by employing the Agilent 2100 bioanalyzer (Agilent Technologies, Waldbronn, Germany) and the High Sensitivity DNA Assay Kit (Agilent Technologies, Waldbronn, Germany). After verification of quality, 56 amplicons from different cell pools were pooled in equal shares and mixed with 10% PhiX before being run. Reads were mapped employing the Bismark software^32^ which uses Bowtie2^33^ for alignment.

### Computational analysis

Statistical analysis was performed with the JMP10 software (JMP®10.0.1 Release: 2, 64-bit edition; SAS Institute Inc.). The non-parametric Stell-Dwass test was employed for all-pairs multiple comparisons with non-normally distributed data. The Tukey HSD test was applied for all-pairs multiple comparisons with normally distributed data. Normal distribution was assessed with the Shapiro-Wilk test and visual inspection of the normal-quantile plot. Outlier analysis was performed with jackknife.

Transcription factor binding sites within the hCMV-MIE were identified with the PROMO 3.0 software for *Cricetidae* species using the TRANSFAC database version 8.3.

## Additional Information

**How to cite this article**: Moritz, B. *et al.* CMV promoter mutants with a reduced propensity to productivity loss in CHO cells. *Sci. Rep.* **5**, 16952; doi: 10.1038/srep16952 (2015).

## Supplementary Material

Supplementary Information
