# Supplementary material for: CMV promoter mutants with a reduced propensity to productivity loss in CHO cells
Source: Sci Rep. 2015 Nov 19;5:16952. doi: 10.1038/srep16952 (PMC4652263; doi:10.1038/srep16952)
Supplement: Supplementary Information [file srep16952-s1.doc]

**Supplementary Information**

**CMV promoter mutants with a reduced propensity to productivity loss in CHO cells**

Benjamin Moritz*, Peter B. Becker+, Ulrich Göpfert*

*Roche Pharmaceutical Research and Early Development, Large Molecule Research, Roche Innovation Center Penzberg, Germany

+ Biomedical Center and Center for Integrated Protein Science Munich, Ludwig Maximilian University, Munich, Germany

Corresponding author:

Ulrich Göpfert

Roche Pharmaceutical Research and Early Development, Large Molecule Research, Roche Innovation Center Penzberg

Roche Diagnostics GmbH

Nonnenwald 2

82377 Penzberg / Germany

Phone: +49 8856 60 4033

Fax: +49 8856 60 2659

E-mail: ulrich.goepfert@roche.com

Supplementary Figure S1

**
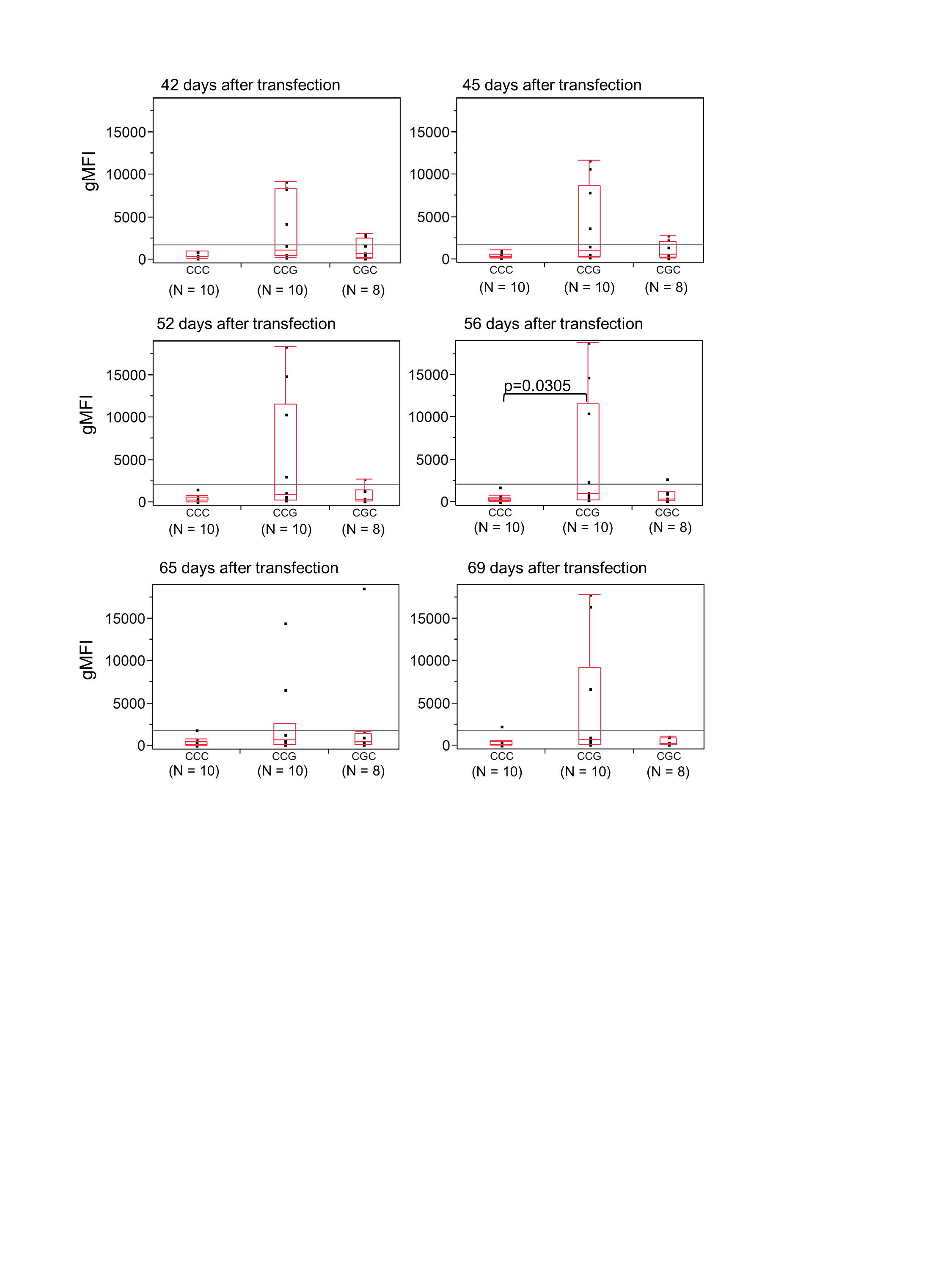
**

**eGFP expression of permanently transfected CHO cell pools at different time points from day 42 to day 69 after transfection**

eGFP expression was quantified by FACS and the geometrical mean of the fluorescence intensity (gMFI) was plotted for each cell pool. The identity of the promoter variants and the number (N) of independent cell pools is indicated below the x-axis. The upper and the lower ends of the boxes represent the first and the third quartile of each group. The ends of the whiskers represent the lowest and highest values still within the 1.5fold interquartile range. The grey line indicates the overall mean of the gMFI values. Differences between the groups were tested for significance with the Steel-Dwass test (α=0.05). One significant effect was detected on day 56 and is indicated by the p-value.

Supplementary Figure S2


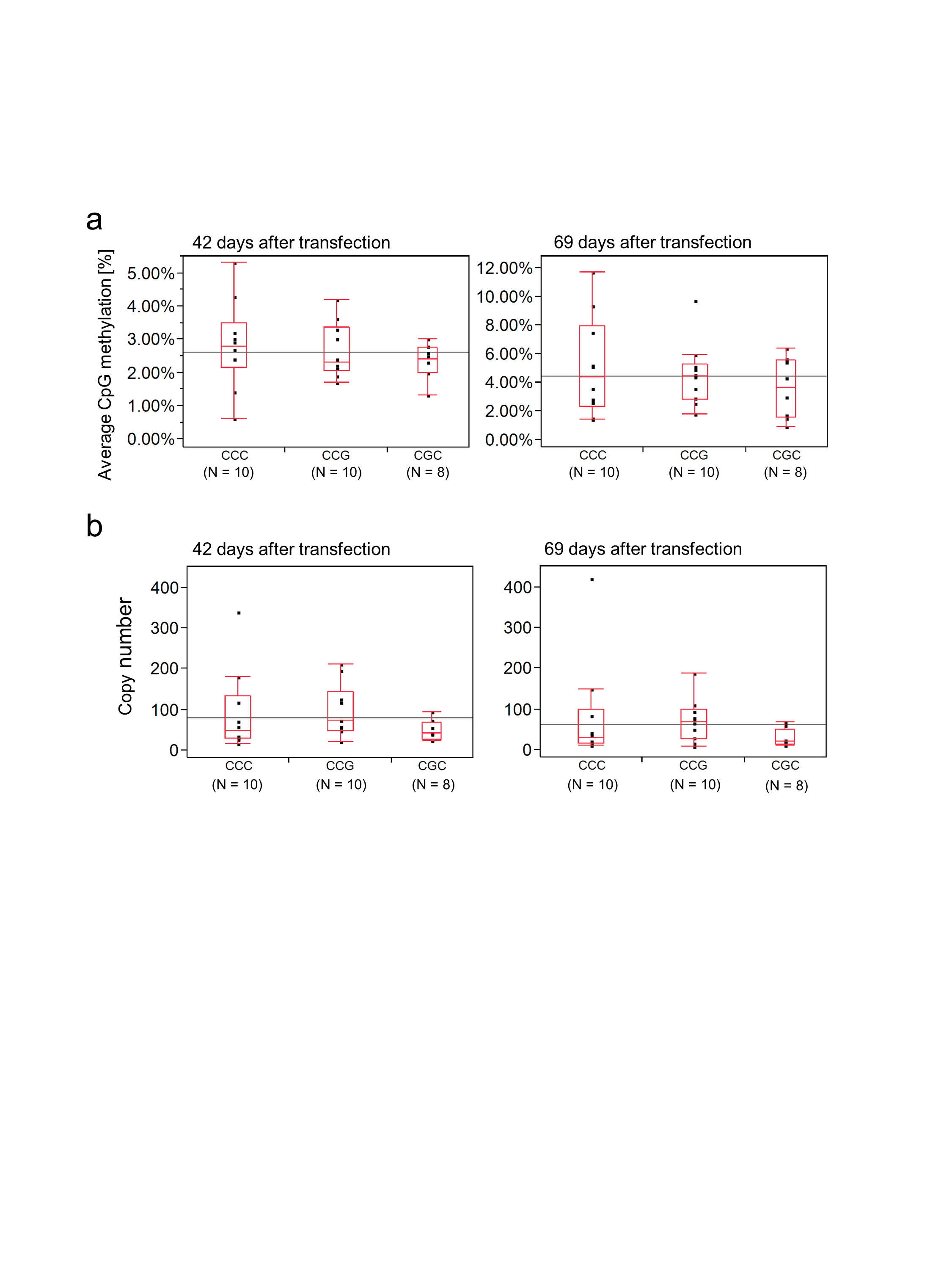


**Overall hCMV-MIE methylation levels and transgene copy numbers of eGFP expressing CHO pools**

Average methylation levels of all hCMV-MIE CpG sites **(a)** and average number of transgene copies **(b)** in permanently transfected eGFP expressing CHO pools 42 (left panels) and 69 days (right panels) after transfection. The identity of the promoter variants and the number (N) of independent cell pools is indicated below the x-axis. The upper and the lower ends of the boxes represent the first and the third quartile of each group. The ends of the whiskers represent the lowest and highest values still within the 1.5fold interquartile range. The grey line indicates the overall mean of the y-variable in the diagram.
